# Identification and functional characterization of fish IL-17 receptors suggest important roles in the response to nodavirus infection

**DOI:** 10.1007/s42995-024-00225-1

**Published:** 2024-04-18

**Authors:** Carmen González-Fernández, Miguel A. García-Álvarez, Alberto Cuesta

**Affiliations:** 1 Department of Cell Biology and Histology, Faculty of Biology, University of Murcia, 30100 Murcia, Spain; 2 Laboratoire d’écotoxicologie, Centre de Lyon-Villeurbanne, INRAE, UR RiverLy, 69625 Villeurbanne, France

**Keywords:** Th17 cells, IL-17 receptors, Immunity, Teleost fish, Nodavirus (NNV), Aquaculture

## Abstract

Th17 is a lymphocyte T helper (Th) subpopulation relevant in the control and regulation of the immune response characterized by the production of interleukin (IL)-17. This crucial cytokine family acts through their binding to the IL-17 receptors (IL-17R), having up to six members. Although the biology of fish Th17 is well-recognized, the molecular and functional characterization of IL-17 and IL-17R has been limited. Thus, our aim was to identify and characterize the IL-17R repertory and regulation in the two main Mediterranean cultured fish species, the gilthead seabream (*Sparus aurata*) and the European sea bass (*Dicentrarchus labrax*). Our in silico results showed the clear identification of six members in each fish species, from IL-17RA to IL-17RE-like, with well-conserved gene structure and protein domains with their human orthologues. All of them showed wide and constitutive transcription in naïve tissues but with highest levels in mucosal tissues, namely skin, gill or intestine. In leucocytes, T mitogens showed the strongest up-regulation in most of the *il17 receptors* though *il17ra* resulted in inhibition by most stimulants. Interestingly, in vivo nodavirus infection resulted in alterations on the transcription of *il17 receptors*. While nodavirus infection led to some increments in the *il17ra*, *il17rb*, *il17rc* and *il17rd* transcripts in the susceptible European sea bass, many down-regulations were observed in the resistant gilthead seabream. Our data identify the presence and conservation of six coding IL-17R in gilthead seabream and European sea bass as well as their differential regulation in vitro and upon nodavirus infection.

**Supplementary Information:**

The online version contains supplementary material available at 10.1007/s42995-024-00225-1.

## Introduction

Innate immune system recognizes and interacts with common molecules present in pathogens, referred to as pathogen-associated molecular patterns (PAMPs), through pattern-recognition receptors (PRRs). This interaction triggers the production of a cascade of immune molecules, mainly proinflammatory cytokines, chemokines and antimicrobial peptides (AMPs) (Akira et al. 2006). To coordinate the functions between the immune and nonimmune cells, and their products, the presence and role of T helper (Th) lymphocytes (CD4 +) is critical in vertebrate’s immunity. Divided into several populations, apart from the classical Th1 and Th2 cells, the existence and knowledge about other Th populations, including T regulatory cells (Treg), follicular helper T cells (Tfh), the potentially distinct T helper 9 (Th9), the regulatory type 1 cells (Tr1), or the Th17 cells, is emerging. Th17 has been revealed as a very relevant cell subpopulation, defined by its activation by interleukin (IL)-23 and secretion of IL-17, among other cytokines (Park et al. 2005). IL-17 cytokines, with members from A to F, are mainly produced by Th17 cells and display functions in the immunological response toward pathogens, including the mucosal tissues, autoimmune disorders, allergy, or inflammatory insults among others, by their binding to IL-17 receptors (IL-17R). Five members of the IL-17R family were initially discovered (Moseley et al. 2003), and named IL-17RA to IL-17RE, though a sixth one, IL-17RE-like, has been also described (Wu et al. 2011). They form heterodimers to bind IL-17 ligands: IL-17RA may dimer with IL-17RC for IL-17A and F, with IL-17RB for IL-17E, and with IL-17RD for unknown ligand; or homodimers of IL-17RB for IL-17B and IL-17RE for IL-17C. However, no clear receptor for IL-17D nor ligand for IL-17RD and IL-17RE-like have been identified so far in any vertebrates. The most studied is the IL-17RA and IL-17A/F biology, but are not completely understood. Therefore, further studies are needed to ascertain the precise roles of IL-17 ligands and receptors in immunity and disease.

Fish are excellent model animals to study the evolution of immunity due to the first apparition of lymphocytes and the adaptive response. Functional and genomic studies demonstrated that fish possess Th17 cells because both IL-17 ligands and IL-17Rs have been documented though they may be less evolved and specialized than their mammalian counterparts (Ashfaq et al. 2019). Unfortunately, the existence of a true and unique Th17 cell population, and its distribution and regulatory functions remain unexplored. Regarding IL-17Rs, one-to-six members have been identified in many species, including Japanese pufferfish (*Takifugu rubripes*), zebrafish (*Danio rerio*) (Fürthauer et al. 2002; Wu et al. 2011), rainbow trout (*Oncorhynchus mykiss*) (Monte et al. 2013), channel catfish (*Ictalurus punctatus*) (Wang et al. 2014), large yellow croaker (*Larimichthys crocea*) (Ding et al. 2016), Japanese medaka (*Oryzias latipes*) (Harada et al. 2021), orange-spotted grouper (*Epinephelus coioides*) (Jiang et al. 2017), spotted sea bass (*Lateolabrax maculatus*) (Mao et al. 2020), or turbot (*Scophthalmus maximus*) (Xue et al. 2021). These studies have provided evidence of good domain conservation as well as an up-regulation in immune and mucosal tissues upon bacterial challenge. Regarding parasitic diseases, the IL-17RA coding gene was down-regulated in grouper upon infection with *Cryptocaryon irritans* (Jiang et al. 2017), but not the rest of IL-17 receptors. This occurred also for rainbow trout infected with *Tetracapsuloides bryosalmonae* (Monte et al. 2013). However, only one study has evaluated the regulation during viral infections, describing that IL-17RA is not altered by infection of trout with viral hemorrhagic septicaemia virus (VHSV) (Monte et al. 2013). In this regard, previous RNA-seq analysis in our laboratory identified the up-regulation of IL-17RA and IL-17RC coding genes in European sea bass (*Dicentrarchus labrax*) leucocytes or cell line against nodavirus (NNV) (Chaves-Pozo et al. 2017, 2019). Similarly, few studies have confirmed that viral infection regulates fish IL-17 ligands (Chi and Sun 2015; González-Fernández et al. 2020; Monte et al. 2013) though transcriptomic analysis failed to describe the viral regulation of IL-17Rs among the differentially expressed genes (Labella et al. 2018; Lama et al. 2020; Peruzza et al. 2021; Tso and Lu 2018). In addition, several binding assays have tried to reveal the IL-17 ligand and receptor interactions. Thus, IL-17A/F proteins bind to IL-17RC in Japanese medaka (Harada et al. 2021), whereas IL-17D did to IL-17RA in lamprey (Han et al. 2015).

Understanding the biology of fish Th17 populations would increase our knowledge for both evolutionary and practical purposes. Thus, we aimed for the first time, to achieve the identification and transcriptional evaluation of IL-17 receptors in the gilthead seabream and European sea bass, which are bony fish species of great economic importance for the Mediterranean aquaculture industry (APROMAR 2023). These species are severely affected by numerous pathogens, mainly viruses, bacteria, and parasites, causing serious infectious diseases and economic losses in the fish farms (Muniesa et al. 2020). Among them, nervous necrosis virus (NNV), which causes viral encephalopathy and retinopathy, affects many seabream and sea bass farms (Muniesa et al. 2020), and leads to large-scale mortalities and severe losses. NNV replicates in the brain and eye leading to death in most larvae and juveniles although some become resistant and act as reservoirs, spreading the disease. Considering that the susceptibility to NNV has been connected to an exacerbated inflammatory response (Montes et al. 2010) and that the T cell biology (González-Fernández et al. 2021, 2020; Scapigliati et al. 2010) may be altered, deeper characterization of the Th17 cell biology is vital to understand NNV-fish interactions. Therefore, we aimed for the first time to identify all the IL-17Rs in gilthead seabream and European sea bass and their regulation by NNV infection. Taking all these into consideration, we have identified six IL-17 receptors in gilthead seabream and European sea bass in silico*,* and evaluated their gene expression in tissues from naïve specimens, their regulation in head-kidney leucocytes (HKLs) upon in vitro stimulation as well as in the brain and head-kidney upon NNV infection in vivo. Results are discussed to ascertain their role in fish immunity and regulation during viral infection.

## Materials and methods

### Bioinformatic analysis

European sea bass and gilthead seabream sequences for IL-17 receptors were searched in NCBI, Ensembl and RNA-seq databases. Putative proteins were predicted from the mRNA sequences within the ExPASy Molecular Biology server (http://us.expasy.org). Homology analysis of the IL-17 receptor protein sequences was performed with the BLAST algorithm (http://blast.ncbi.nlm.nih.gov/Blast.cgi) and a phylogenetic tree was constructed by the MEGA 11.0 program with amino acid sequences and bootstrap value set to 10,000 replicates (Tamura et al. 2021). Protein domains were identified by Pfam, Gene3D or PROSITE databases and drawn by the MyDomains—Image Creator tool (https://prosite.expasy.org/mydomains/).

### Experimental design and sampling

Samples from previous experiments (Chaves-Pozo et al. 2012; Esteban et al. 2013) were used according to the 3R’s principle. For constitutive mRNA levels gill, brain, gonad, liver, skin, gut, spleen, and head-kidney (HK), thymus and blood from three healthy naïve fish were sampled and immediately frozen in TRIzol^®^ Reagent (Life Technologies) and kept at – 80 °C.

For the in vitro study, HKLs were obtained from 5 fish specimens and processed independently as previously described (Esteban et al. 2013). One million HKLs/mL were placed into 48-well microtiter plates (Nunc) and incubated at 22 °C during 24 h with: culture L-15 medium (control treatment), 5 μg/mL lipopolysaccharide (LPS; Sigma-Aldrich), 5 μg/mL concanavalin A (ConA; Sigma-Aldrich), 50 μg/mL synthetic unmethylated cytosine-phosphodiester-guanosine oligodeoxynucleotide 1668 (CpG ODN; sequence 5ʹ-TCCATGACGTTCCTGATGCT-3ʹ; Eurogentec), 10 μg/mL phytohemagglutinin (PHA; Sigma-Aldrich), 25 μg/mL Poly I:C (pI:C; Sigma-Aldrich), 10^8^ bacteria/mL of *Vibrio anguillarum* (Va) or *Photobacterium damselae* (Pd) heat-killed bacteria, and 10^6^ TCID_50_ NNV/mL. Afterward, HKLs were washed with phosphate-buffered saline (PBS) and conserved in TRIzol^®^ Reagent at -80 °C.

For the in vivo infection with NNV, thirty gilthead seabream and European sea bass (125 ± 25 and 305 ± 77 g body weight, respectively) specimens were injected intramuscularly with 100 µL of culture medium (Mock) with or without 10^6^ TCID_50_/fish of NNV (strain 411/96, genotype RGNNV) (Chaves-Pozo et al. 2012). At 1, 7, and 15 days post-infection, brain and HK tissues of each fish (*n* = 4–6 fish) were extracted and immediately frozen in TRIzol® Reagent and kept at – 80 °C.

### Real-time PCR analysis

Total RNA was isolated from TRIzol^®^ Reagent frozen samples following the manufacturer's instructions. Contaminating genomic DNA was digested with DNAse I (Promega) and the first strand of cDNA synthesized by reverse transcription using the Superscript III (Life Technologies) according to the manufacturer's instruction. Real-time PCR was performed using the 7500 Fast Real-Time PCR System (Roche Applied Science) and SYBR Green PCR Core Reagents (Applied Biosystems). Reaction mixtures were incubated at 95 °C for 10 min, followed by 40 cycles of 15 s at 95 °C, 1 min at 60 °C, and finally 15 s at 95 °C, 1 min at 60 °C and 15 s at 95 °C. Gene expression was corrected by the geometric mean of the elongation factor 1 alpha (*ef1a*) and ribosomal 18S (*rps18*) gene expressions. Relative mRNA quantities of the target in each sample were normalized to the expression of the reference genes (Livak and Schmittgen 2001). Primers are listed in Supplementary Table S1. Negative controls with no sample were always included in the reactions.

### Statistical analysis

Statistical analysis was performed using SPSS Statistics 26 and graphs done by GraphPad Prism 8 software. Differences were considered significant at *P* < 0.05. Significant differences in gene expression in the in vitro treatments of HKLs were determined by one-way ANOVA followed by Tukey's post hoc test if required. Normality and homogeneity of variances of the data distribution were tested by standardized skewness and standardized kurtosis and Levene's test, respectively. Additionally, Student *t* test was used to compare between mock- and NNV-infected fish. Non-parametric Pearson correlation test was applied to identify potential relations among gene expression.

## Results

### Identification of IL-17 receptors and in silico analysis

**Fig. 1:**
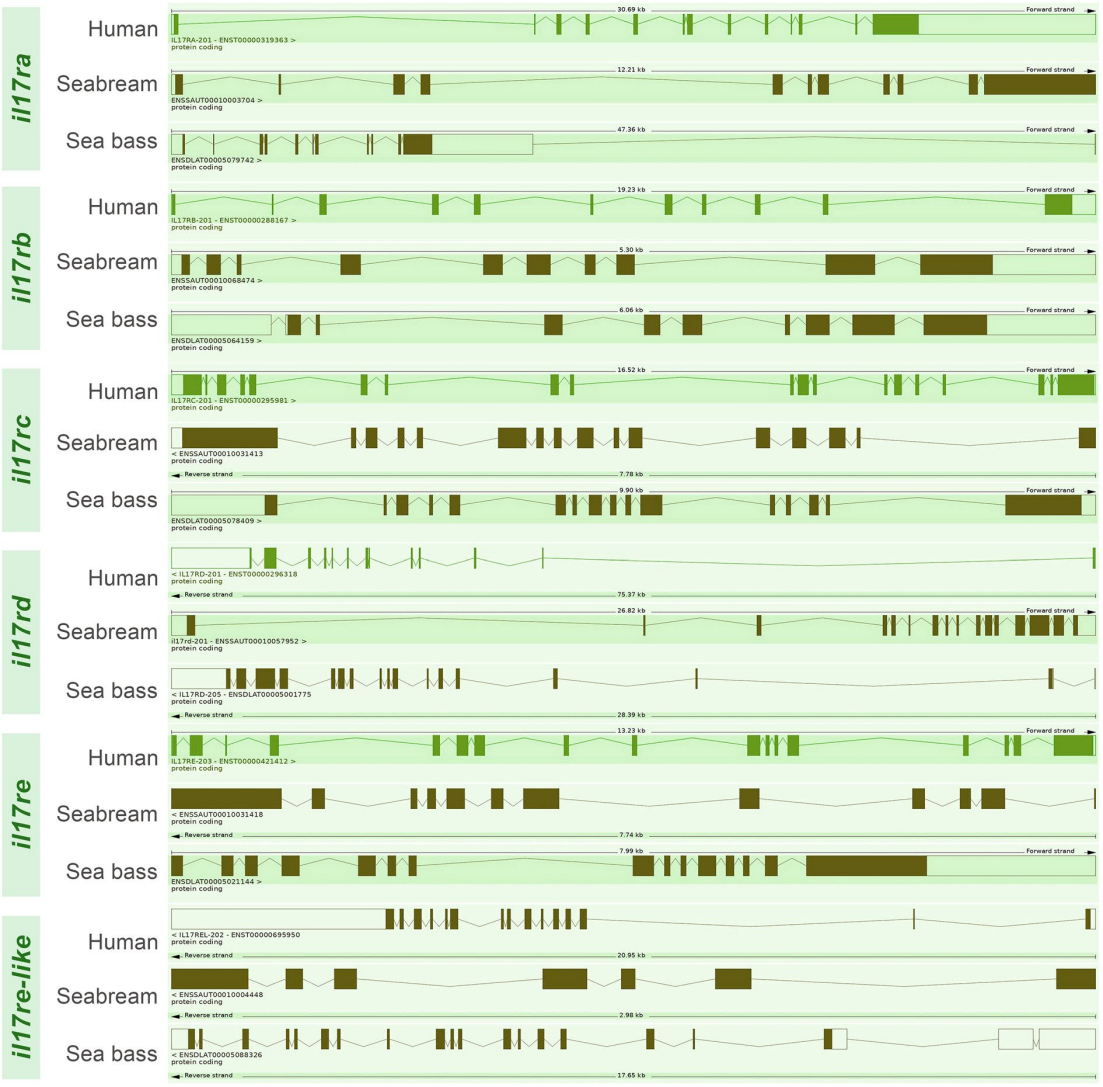
Genomic organization of IL-17 receptor-coding genes. Sequences were retrieved from the Ensembl database. Gene length and accession numbers are indicated. *Exon* boxes, *intron* lines

From different genomic databases, we found six IL-17 receptor coding genes in both gilthead seabream and European sea bass, which were related to their human orthologues, and named from IL-17RA to RE, and IL-17RE-like. Genomic length and exon/intron organization are quite variable. From *il17ra* to *il17re-like* genes, the exon number for the coding sequences is of 11, 10, 16, 16, 12, and 7 for seabream and of 11, 9, 16, 16, 15, and 18 for sea bass, respectively (Fig. 1). The number of exons in their human orthologues is of 13, 11, 19, 13, 17, and 15, respectively. All the genes contained an open reading frame that would generate a full-length putative receptor, though only the seabream IL-17RE-like shows partial sequence. However, incomplete and lack of in-depth genome sequencing and annotation for these fish species might lead to some uncertainties.

**Fig. 2:**
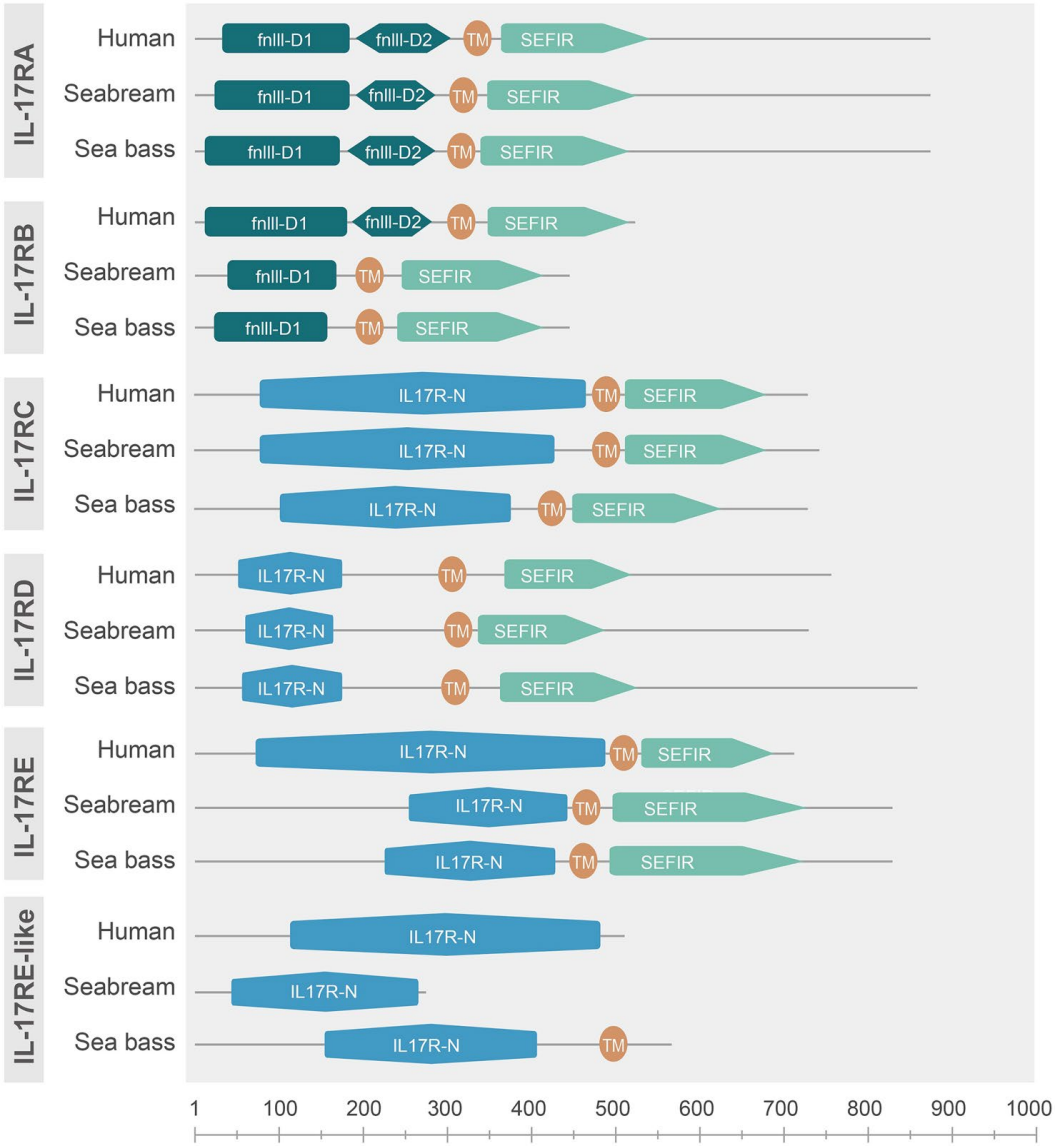
Protein domains identified in the human, gilthead seabream and European sea bass IL-17 receptor orthologues. Domains are presented in proximate length and position according to predictions

Identified genes were translated to putative proteins. Seabream IL-17RE-like sequence is truncated and IL-17RB is the shortest IL-17 receptor. Fish IL-17 receptors were annealed with their respective human orthologues (Supplementary Fig. S1). Similarity between fish proteins varied from 68 to 90% between whole receptors, being maximum for IL-17RD, whereas this was of 39% for IL-17RE-like due to the seabream partial sequence. This similarity against human proteins dropped to 17–58%, resulting again with the highest for IL-17RD (Supplementary Fig. S1). Protein domains and architecture of the putative receptors were predicted using different databases. First, human, seabream and sea bass orthologues of IL-17RA, IL-17RB, IL-17RC, IL-17RD, and IL-17RE clearly showed a well-conserved transmembrane region, whereas this region was not so clearly identified in IL-17RE-like proteins (Fig. 2). In all cases, an IL-17 receptor-like domain (IPR039465) was identified expanding most of the protein length, but other relevant domains were also detected. IL-17RA to IL-17RE contained a cytosolic SEFIR (SEF/IL-17R) domain (IPR013568) quite well conserved in length and position, but this is lacking in IL-17RE-like (Fig. 2). In the N-terminal and extracellular part, all the IL-17RA show good conservation with two IL-17R fibronectin III domains, fnIII_D1 and fnIII_D2 (Fig. 2). For IL-17RB, all of them showed the fnIII_D1 domain, whereas seabream and sea bass lacked the fnIII_D2 domain present in humans (Fig. 2). IL-17RC, IL-17RE, and IL-17RE-like show the presence of the conserved IL-17 receptor C/E N-terminal domain (IPR027841), whereas IL-17RD contains a related domain, named the IL-17 receptor D N-terminal domain (IPR031951) (Fig. 2).

**Fig. 3:**
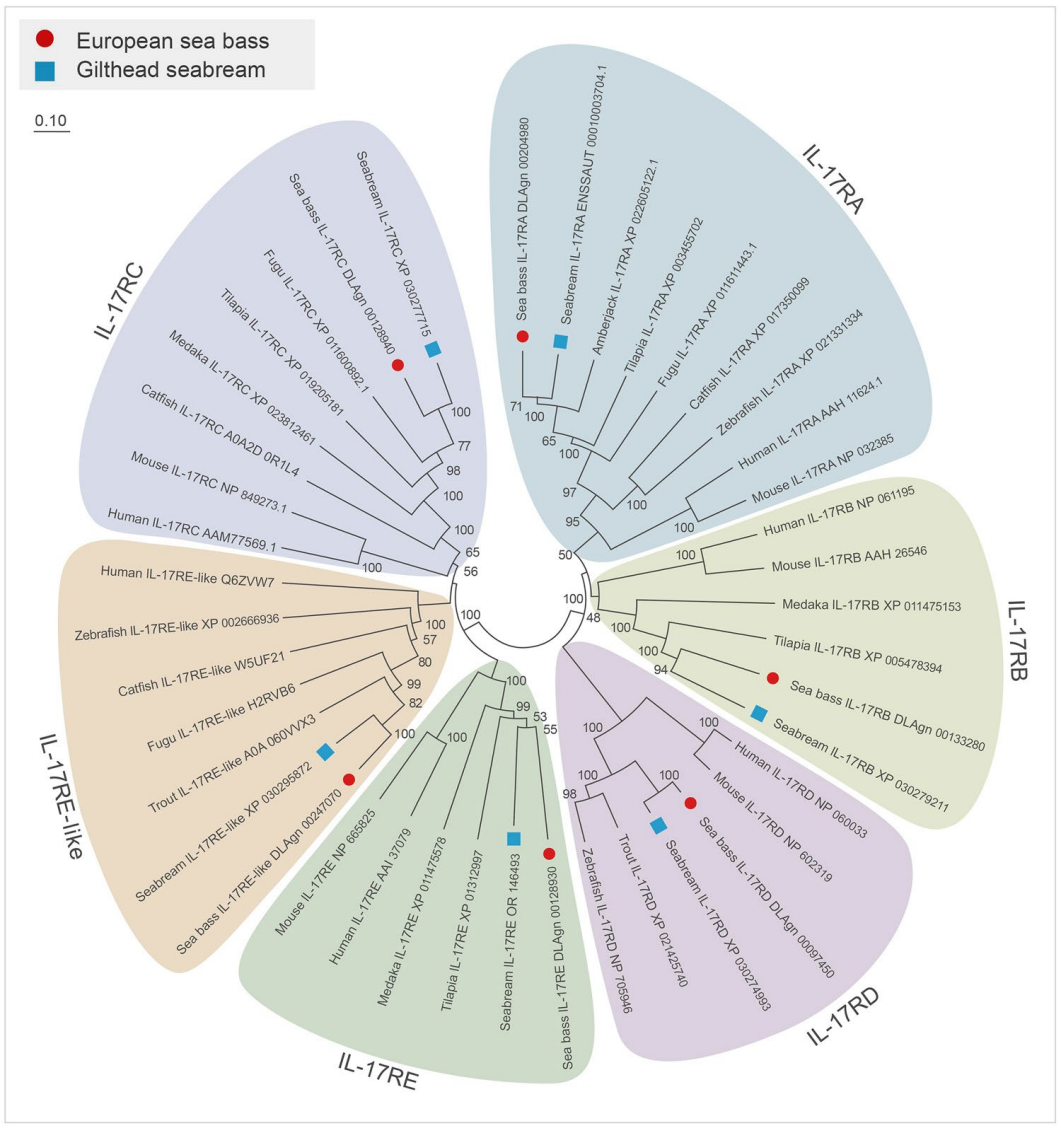
Phylogenetic tree with gilthead seabream and European sea bass IL-17 receptors and orthologues was constructed using the Neighbor-Joining method, where genetic distances were calculated based on protein differences (p-distance) with pairwise deletion. The number of each node represents the percentage of bootstrapping after 10,000 replications. GenBank accession numbers are shown. Protein clades are indicated in different colors

The phylogenetic tree was constructed, and revealed the existence of 6 clades, each containing the corresponding fish and mammalian IL-17R orthologues (Fig. 3), which were grouped into two main clades, the first comprising IL-17RA, IL-17RB, and IL-17RD proteins and the second with IL-17RC, IL-17RE, and IL-17RE-like.

### Tissue distribution of IL-17R transcripts

**Fig. 4:**
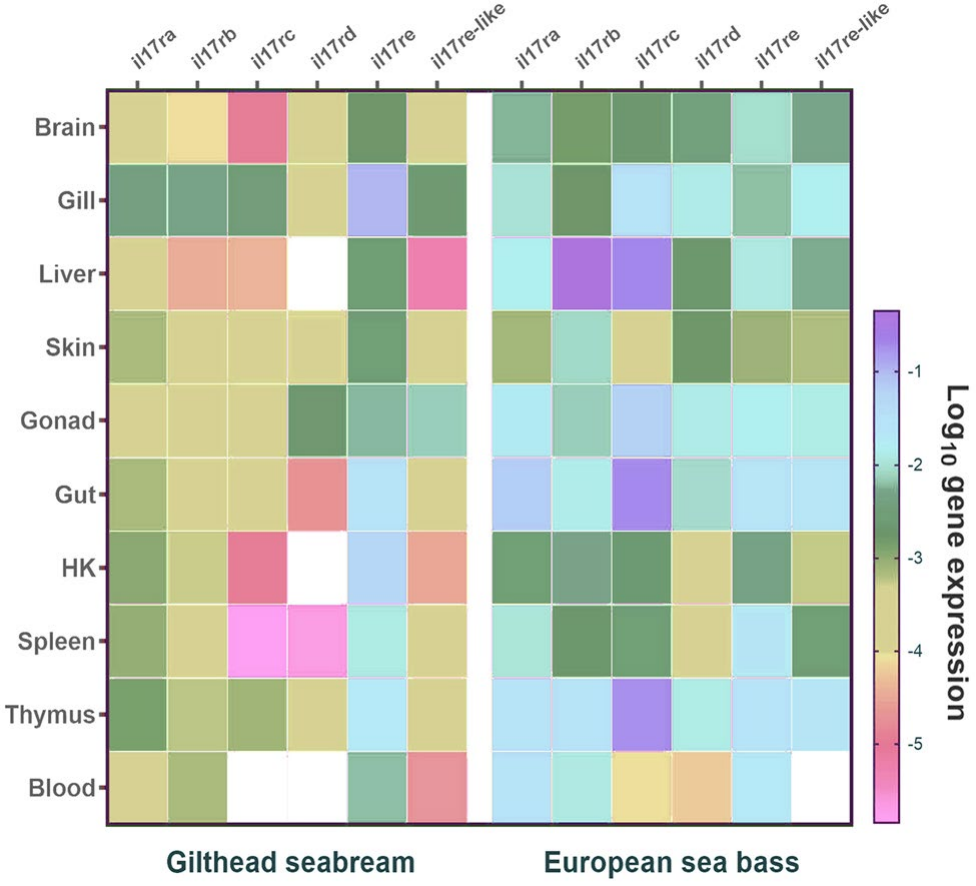
Heatmap showing the mRNA levels of *il17 receptor* genes in different organs from naïve gilthead seabream and European sea bass specimens. Data are shown as log of mean (*n* = 3) target gene expression relative to the expression of endogenous control *ef1a* and *rps18* genes

Distribution of *il17r* genes expression was studied under naïve conditions in seabream and sea bass tissues. Expression data showed that they were ubiquitously distributed but their expression was very low, being usually higher in sea bass than in seabream tissues (Fig. 4). Regarding the seabream, gill was the tissue with the highest transcription for most of the receptors, whereas HK, spleen, blood, and liver showed low transcription levels. The *il17re* of seabream showed the highest expression profile in the tissues (Fig. 4). Regarding sea bass, liver and gut presented the greatest transcription levels in general, whereas HK, spleen, and blood revealed the lowest (Fig. 4). Sea bass *il17rb* and *il17rc* were the most expressed genes in the sea bass tissues.

### *Il17r* gene regulation in head-kidney leucocytes in vitro

**Fig. 5:**
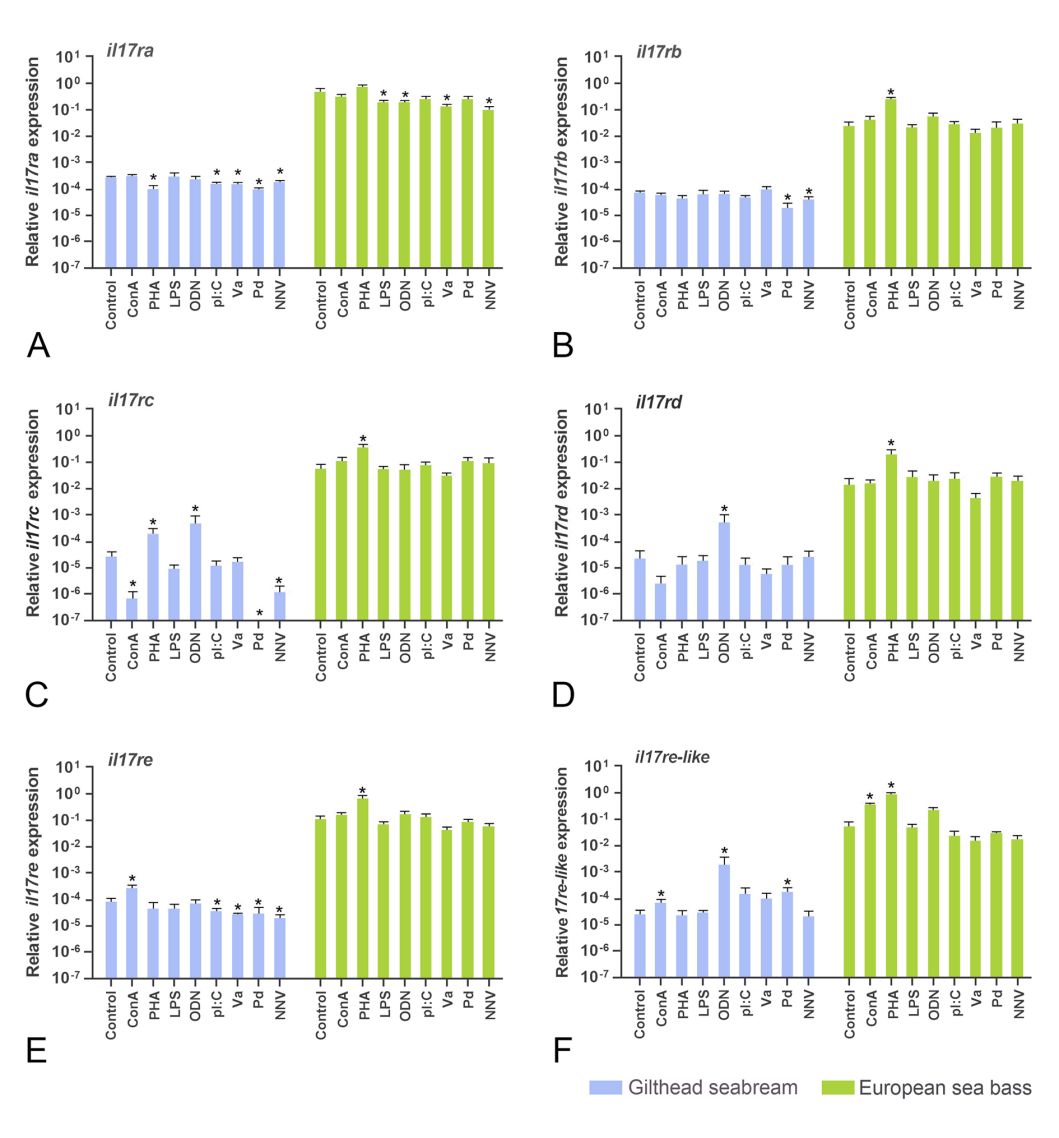
Relative gene expression of *il17 receptors* in the gilthead seabream and European sea bass head-kidney leucocytes incubated for 4 h with culture medium (Control), 5 μg/mL concanavalin A (ConA), 5 μg/mL lipopolysaccharide (LPS), 10 μg/mL phytohemagglutinin (PHA), 50 μg/mL synthetic unmethylated cytosine-phosphodiester-guanosine oligodeoxynucleotide 1668 (ODN), 25 μg/mL PolyI:C (pI:C), 10^8^ heat-killed *Vibrio anguillarum* (Va) or *Photobacterium damselae* (Pd) bacteria/mL, and 10^6^ TCID_50_ nodavirus NNV/mL. Data are presented as means (*n* = 5) ± SEM relative to the expression of the endogenous controls. Asterisks denote differences in expression with the control (ANOVA, *P* < 0.05)

We evaluated the *il17r* gene expression upon stimulation of HKLs (Fig. 5). As seen above, sea bass HKLs showed higher transcription than those of seabream. For seabream HKLs, poly I:C, both bacteria and NNV reduced *il17ra* (Fig. 5A) and *il17re* (Fig. 5E) transcription significantly, whereas NNV reduced the expression of *il17rb* (Fig. 5B) and *il17rc*, also reduced by the Pd organism (Fig. 5C). T cell mitogen PHA up-regulated the expression of *il17rc* but decreased that of *il17ra*, whereas ConA up-regulated the transcription of *il17re* and *il17re-like* but down-regulated *il17rc* (Fig. 5). Interestingly, ODN treatment up-regulated the expression of *il17rc*, *il17rd* and *il17re-like* genes in seabream HKLs. Conversely, sea bass HKLs suffered few significant regulations in IL-17R coding genes. Thus, treatment with PHA up-regulated the transcription of *il17rb*, *il17rc*, *il17rd* and *il17re*, whereas both PHA and ConA did so with *il17re-like* (Fig. 5). In addition, *il17ra* transcription was significantly reduced by LPS, ODN, the Va culture and NNV (Fig. 5).

### Nodavirus infection altered the *il17r* transcription, mainly in the brain of European sea bass

**Fig. 6:**
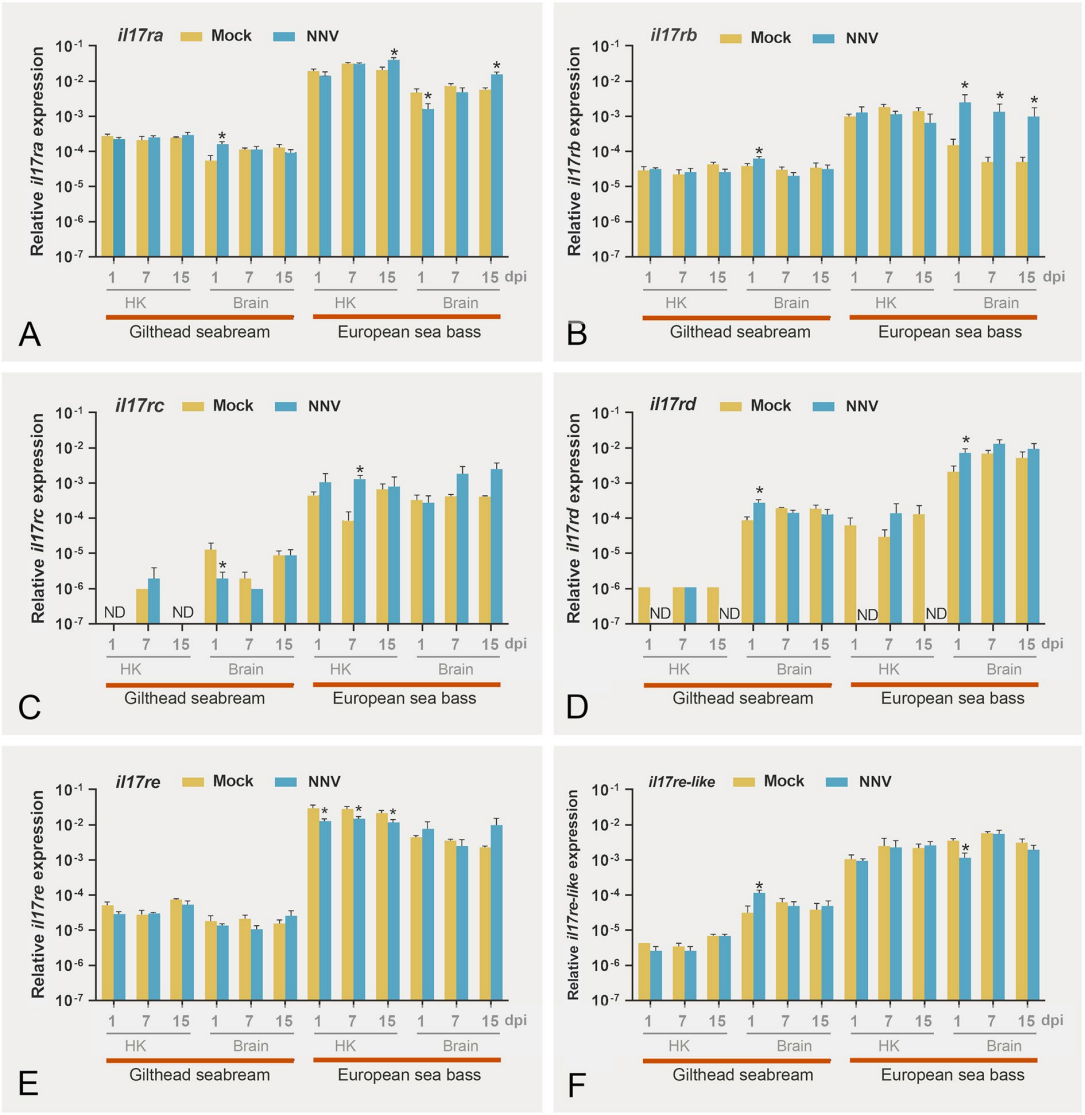
Relative gene expression of *il17 receptors* in the head-kidney and brain of gilthead seabream and European sea bass specimens intramuscularly injected with 100 µL of culture medium alone (Mock) or containing 10^6^ TCID_50_/fish of nodavirus (NNV). Data are presented as means (*n* = 4–6) ± SEM relative to the expression of the endogenous controls. Asterisks denote significant differences with the control group (Student *t* test, *P* < 0.05). *ND* undetected

NNV infection resulted in a 47% survival of European sea bass, whereas gilthead seabream were completely resistant, and mortalities were not recorded (Chaves-Pozo et al. 2012). Therefore, we evaluated the *il17r* transcription in the hematopoietic and the NNV target tissues, the HK and the brain, respectively, as well as in the NNV resistant and susceptible species, gilthead seabream and European sea bass, respectively. In gilthead seabream, the expression profile in the HK was unaffected but, in the brain, *il17rb*, *il17rd*, and *il17re-like* were up-regulated, whereas *il17rc* was down-regulated to a significant extent at 1-day post-infection (dpi) (Fig. 6). In European sea bass, transcription of *il17ra* and *il17rc* was increased in the HK at 15 and 7 dpi, respectively. However, the HK expression of *il17rd* at 1 and 15 dpi was completely blocked (Fig. 6D), whereas that of *il17re* was significantly reduced at all the infection times (Fig. 6E). In sea bass brain, *il17ra* and *il17re-like* were significantly decreased at 1dpi, whereas *il17rb* levels were increased at all the infection times, and *il17ra* and *il17rd* did at 15 and 1 dpi, respectively (Fig. 6).

### Correlation analysis of *il17* ligands and receptors transcripts

We tried to detect potential correlations between the transcription of *il117* receptors and ligands (unpublished for seabream; González-Fernández et al. 2020 for sea bass) in the same samples. In general, most of the correlations between *il17* ligands and receptors were positive (Tables 1 and 2). In seabream, only few strong and significant correlations were found: *il17a/f1* with *il17d* and *il17re-like*, *il17a/f2* with *il17a/f3*, *il17d* with *il17rc* and *il17re-like*, *il17ra* with *il17re*, and *il17rb* with *il17rd* (Table 1). In the case of European sea bass, however, higher, and more significant correlations were observed (Table 2). Regarding the *il17* ligands, although some strong correlations were detected for the transcription of *il17a/f2* and i*l17d*, it is noteworthy that *il17c2* did so with most of the ligands and receptors (Table 2). Strikingly, all the sea bass IL-17 receptors showed significant and strong correlations among them (Table 2) in sharp contrast to what was observed in seabream (Table 1).

**Table 1:** Correlation matrix for gilthead seabream transcripts of *il17* ligands and receptors in naïve tissues, stimulated head-kidney leucocytes and nodavirus-infected fish

		*il17a/f2*	*il17a/f3*	*il17c2*	*il17d*	*il17ra*	*il17rb*	*il17rc*	*il17rd*	*il17re*	*il17re-like*
*il17a/f1*	Pearson	– 0.029	– 0.029	– 0.019	**0.630**	0.257	0.136	0.413	0.039	0.104	**0.902**
	*P* value	*0.745*	*0.745*	*0.829*	***0.000***	***0.003***	*0.124*	***0.000***	*0.659*	*0.243*	***0.000***
*il17a/f2*	Pearson	1	**1.000**	– 0.007	– 0.03	0.019	– 0.025	– 0.024	– 0.025	0.02	– 0.057
	*P* value		***0.000***	*0.937*	*0.735*	*0.833*	*0.775*	*0.812*	*0.781*	*0.822*	*0.518*
*il17a/f3*	Pearson		1	– 0.007	– 0.03	0.019	– 0.025	– 0.024	– 0.025	0.02	– 0.057
	*P* value			*0.937*	*0.735*	*0.833*	*0.775*	*0.812*	*0.781*	*0.822*	*0.518*
*il17c2*	Pearson			1	– 0.023	– 0.008	– 0.023	– 0.02	– 0.028	– 0.03	– 0.03
	*P* value				*0.795*	*0.927*	*0.797*	*0.845*	*0.749*	*0.739*	*0.74*
*il17d*	Pearson				1	0.121	0.089	**0.692**	0.02	0.035	**0.765**
	*P* value					*0.171*	*0.314*	***0.000***	*0.818*	*0.697*	***0.000***
*il17ra*	Pearson					1	0.311	0.094	0.376	**0.723**	0.255
	*P* value						***0.000***	*0.346*	***0.000***	***0.000***	***0.004***
*il17rb*	Pearson						1	0.218	**0.914**	0.075	0.099
	*P* value							***0.028***	***0.000***	*0.398*	*0.266*
*il17rc*	Pearson							1	0.224	– 0.015	0.479
	*P* value								***0.024***	*0.881*	***0.000***
*il17rd*	Pearson								1	0.221	0.004
	*P* value									***0.012***	*0.961*
*il17re*	Pearson									1	0.144
	*P* value										*0.104*

Significant correlations and Pearson indexes > 0.5 are highlighted in bold. Transcription of *il17* ligands is unpublished

**Table 2:** Correlation matrix for European sea bass transcripts of *il17* ligands and receptors in naïve tissues, stimulated head-kidney leucocytes and nodavirus-infected fish

		*il17a/f2*	*il17a/f3*	*il17c1*	*il17c2*	*il17d*	*il17n*	*il17ra*	*il17rb*	*il17rc*	*il17rd*	*il17re*	*il17re-like*
*il17a/f1*	Pearson	0.208	0.033	0.064	0.481	0.458	0.294	0.411	0.258	**0.579**	0.406	0.422	0.394
	*P* value	***0.025***	*0.723*	*0.592*	***0.000***	***0.000***	***0.001***	***0.000***	***0.005***	***0.000***	***0.000***	***0.000***	***0.000***
*il17a/f2*	Pearson	1	0.042	0.214	**0.762**	**0.811**	**0.628**	0.343	0.141	0.274	0.288	0.229	0.234
	*P* value		*0.658*	*0.071*	***0.000***	***0.000***	***0.000***	***0.000***	*0.133*	***0.003***	***0.002***	***0.014***	***0.012***
*il17a/f3*	Pearson		1	0.037	– 0.030	– 0.017	0.004	0.066	– 0.016	0.032	– 0.009	0.010	– 0.002
	*P* value			*0.759*	*0.748*	*0.860*	*0.964*	*0.485*	*0.864*	*0.736*	*0.928*	*0.918*	*0.986*
*il17c1*	Pearson			1	– 0.099	0.082	0.382	– 0.096	0.020	0.064	– 0.063	– 0.092	0.018
	*P* value				*0.407*	*0.496*	***0.001***	*0.420*	*0.868*	*0.594*	*0.596*	*0.441*	*0.881*
*il17c2*	Pearson				1	**0.923**	**0.681**	**0.585**	0.392	**0.539**	**0.681**	**0.628**	**0.595**
	*P* value					***0.000***	***0.000***	***0.000***	***0.000***	***0.000***	***0.000***	***0.000***	***0.000***
*il17d*	Pearson					1	**0.676**	0.467	0.291	0.383	**0.507**	0.471	0.481
	*P* value						***0.000***	***0.000***	***0.002***	***0.000***	***0.000***	***0.000***	***0.000***
*il17n*	Pearson						1	0.434	0.246	0.359	0.467	0.457	0.380
	*P* value							***0.000***	***0.008***	***0.000***	***0.000***	***0.000***	***0.000***
*il17ra*	Pearson							1	0.490	**0.662**	**0.698**	**0.808**	**0.731**
	*P* value								***0.000***	***0.000***	***0.000***	***0.000***	***0.000***
*il17rb*	Pearson								1	**0.6**	**0.562**	**0.612**	**0.573**
	*P* value									***0.000***	***0.000***	***0.000***	***0.000***
*il17rc*	Pearson									1	**0.741**	**0.757**	**0.656**
	*P* value										***0.000***	***0.000***	***0.000***
*il17rd*	Pearson										1	**0.882**	**0.739**
	*P* value											***0.000***	***0.000***
*il17re*	Pearson											1	**0.899**
	*P* value												***0.000***

Significant correlations and Pearson indexes > 0.5 are highlighted in bold. Transcription of *il17* ligands has been published (González-Fernández et al. 2020)

## Discussion

Fish Th1, Th2, Th17, and Treg cell subpopulations have been clearly identified though they need to be characterized in depth. In fish, naïve Th cells are differentiated to Th17 lymphocytes by transforming growth factor beta (TGF-β) and IL-16 throughout the master transcription factor RORγt (retinoic acid receptor-related orphan receptors gamma). Th17 cells produce and secrete IL-17A/F, IL-21, and IL-22 cytokines playing a major role in the inflammatory response, the response against parasites, and the control of autoimmune disorders (Ashfaq et al. 2019; Tian et al. 2023). Although knowledge of fish IL-17 ligands is in its infancy, much less is known about the IL-17 receptors that mediate their functions. Thus, we have identified six IL-17 receptor coding genes in two marine fish species relevant for Mediterranean aquaculture, i.e., gilthead seabream and European sea bass, and also evaluated their regulation.

### Identification and molecular analysis of IL-17 receptors

The search of gilthead seabream and European sea bass genomes resulted in the identification of six orthologues to human IL-17 receptors. As also evidenced in other vertebrates, they are variable in genomic organization and length though all the putative proteins showed relevant and well-conserved domain architectures. It is remarkable that there is conservation of the following domains: fnIII and SEFIR in IL-17RA and IL-17RB, IL-17R_N and SEFIR in IL-17RC, IL-17RD and IL-17RE, and only the IL-17R_N in IL-17RE-like (reviewed by Gaffen 2009; Wu et al. 2011). Unfortunately, no studies have focused on the relevance or necessity in the functioning of the identified fish IL-17R domains. All the fish studies agree with the identification of the SEFIR domain except for IL-17RE-like. SEFIR domain binding to the Act1 adaptor is necessary to transduce the signaling leading to IL-17 regulation of the inflammatory response (Seon et al. 2006). Thus, we could speculate that all fish IL-17Rs except IL-17RE-like may conserve this interaction and signaling properties though there is a need for confirmation. However, some differences were also detected due to the proper identification of the fnIII domains, which are essential in the dimerization process and binding to the respective ligands (Goepfert et al. 2022). For example, some authors identify extracellular fibronectin III domains in all the human IL-17 receptors (Wu et al. 2011) although others only do so in IL-17RA, IL-17RB and IL-17RD (Ding et al. 2016). All the fish IL-17RA show the presence and good conservation of the two fnIII domains. By contrast, most fish IL-17RB present only one fnIII domain although this is absent in turbot (Xue et al. 2021). Strikingly, more divergence is found for the IL-17RD. We failed to identify these fnIII domains in human, seabream, and sea bass IL-17RD after searching in different databases although they were identified in fish by others (Ding et al. 2016; Xue et al. 2021). For example, the fnIII domains were identified in one turbot IL-17RD form but not in another, in which the IL-17R_D_N domain was detected instead (Xue et al. 2021), as well as in zebrafish and fugu (Wu et al. 2011). In our case, the analysis of seabream and sea bass IL-17RD isoforms using the Ensembl database always identified the IL-17R_D_N domain, and never fnIII. However, the annealing of the extracellular part of seabream and sea bass IL-17RD with the fnIII domains reveals very high similarities. These data would support a homo- or heterodimerization for fish IL-17RA, IL-17RB, and IL-17RD, but experimental evidence is needed. In fact, the phylogenetic analysis clusters IL-17RD with IL-17RA and IL-17RB, with probed fnIII domains, supporting the presence, the conservation, and the functions of this domain. Probably, this is the reason explaining that in a few phylogenetic studies IL-17RA, IL-17RB and IL-17RD do not cluster together. Regarding the presence and the identification of the IL-17R_N domain, most studies in fish identify it in IL-17RC, IL-17RE, and IL-17RE-like, and is the reason they cluster together in phylogenetic trees, which have been considered evolved by duplication from an IL-17RC ancestor (Wu et al. 2011). Therefore, future molecular and bioinformatic analyses should focus on protein organization for IL-17 receptors in order to make stronger biological predictions.

### IL-17R transcription in naïve tissues

Once identified the IL-17R repertoire and molecular structure, we evaluated their transcriptional regulation in an effort to understand their biology. Our data show constitutive and wide tissue distribution or *il17 receptors* being variable with the gene and fish species. Strikingly, tissues where the secondary immune response or first line of defense, such as the mucosal tissues intestine, skin, or gills, show the highest transcriptional profiles, probably because they are the target tissues for the IL-17 ligands produced by Th17 cells. This pattern is similar to those described for several fish species (Ding et al. 2016; Han et al. 2015; Wang et al. 2014; Xue et al. 2021). This is relevant as described in mammals (reviewed by Gaffen 2009) where IL-17A shows the greatest activity on epithelial and endothelial cells, and fibroblasts, with high and correlating IL-17RA levels, being important in the control of inflammation in tissues, such as skin or intestine, and gills. Also, some studies relate the expression of IL-17RB in endocrine tissues, such as liver or kidney and of IL-17RC in non-immune cells of the kidney, prostate, thyroid, liver, and joints though their exact distribution, and the roles are still under discussion. However, the concrete IL-17 receptor expression, pairing, and affinity to IL-17 ligands appear to orchestrate the final Th17 biology and response (reviewed by Gaffen 2009), which has to be also occurring in fish and deserve further investigation. As an example, our data would be in line with this hypothesis because some *il17* ligands show correlation with certain *il17r*.

### IL-17R transcription in stimulated leucocytes

We evaluated the IL-17 receptor genes in the seabream and sea bass HKLs upon stimulation for the first time in fish. Transcription of seabream *il17ra*, *il17rc*, and *il17re* is down-regulated by most stimulants used herein. In contrast, mitogens ConA and/or PHA were able to up-regulate the transcription of most of the sea bass *il17 receptors* suggesting their transcription in T cells, probably Th17 cells. In lamprey, leucocyte distribution demonstrated that lymphocytes are the major producers for IL-17RA, IL-17RD, or IL-17RE/RC, whereas monocytes are for IL-17RE (Han et al. 2015). This is partially in line with our data where receptors are up-regulated by T mitogens. According to the Human Protein Atlas (https://www.proteinatlas.org/), the main leucocytes expressing IL-17 receptors are: IL-17RA in neutrophils; IL-17RB in basophils, Treg and Th2 cells; IL-17RC in monocytes and dendritic cells; IL-17RD in basophils, but with very low transcription; IL-17-RE in Th17 cells; and IL-17RE-like in T cells. Also, our data show that seabream *il17rc* and *il17rd* are increased by ODN, which also increased the innate cell-mediated cytotoxicity (CMC). By contrast, killed bacteria showed down-regulation or no alteration of the seabream or sea bass *il17 receptors* because these killed bacteria produce very little inflammatory response under these conditions. However, in vivo studies in fish clearly point to their up-regulation upon bacterial infection (Ding et al. 2016; Harada et al. 2021; Jiang et al. 2017; Mao et al. 2020; Wang et al. 2014; Xue et al. 2021) based on their role in the inflammatory response in fish.

### Nodavirus regulation of IL-17 receptors

Implications of IL-17R in fish viral response are, however, scarcely evaluated. Thus, trout infection with VHSV failed to alter the *il17ra* transcription (Monte et al. 2013) whereas it was down-regulated in Koi carp infected with Koi herpesvirus (KHV) although the IL-17 pathway was greatly increased (Yang et al. 2022). Regarding NNV, we have searched transcriptomic databases from previous studies on several fish species infected with NNV and did not find the regulation of IL-17R coding genes or the IL-17 pathway (Labella et al. 2018; Lama et al. 2020; Peruzza et al. 2021; Tso and Lu 2018). However, we have already observed that sea bass brain DLB-1 cell line infected with NNV and European sea bass leucocytes during an innate CMC response against NNV-infected cells showed up-regulated *il17ra* and *il17rc* genes (Chaves-Pozo et al. 2017, 2019). These results prompted us to perform this deeper characterization of IL-17 receptors. First, in vitro exposure of leucocytes to NNV, in which the virus does not replicate, led to reduction of *il17ra*, *il17rb*, *il17rc*, and *il17re* in seabream and only of *il17ra* in sea bass, which agrees with the scarce regulation of the IL-17 ligands in the same conditions (González-Fernández et al. 2020). By contrast, during the in vivo infection with NNV, our data show certain regulation of *il17 receptors*, mainly up-regulations of *il17ra*, *il17rb*, or *il17rd* in the European sea bass brain, the target tissue and susceptible species, coinciding with increments of the *il17c1* and *il17d* ligands (González-Fernández et al. 2020). However, some down-regulations are also detected, mainly in gilthead seabream, the resistant species, where the IL-17 ligand genes were not altered. These data agree with those describing an inflammatory response in the brain of sea bass (Montes et al. 2010) but not in seabream, suggesting a role for Th17 cells in the NNV-induced immunity in tissues where the viral replication is high. The *il17ra* is a key signaling player of the IL-17 family because most of the IL-17 family members require it for signaling. However, *il17rb* was the most up-regulated in sea bass brain upon NNV infection. It is known than human IL-17RA could dimer with either IL-17RB or IL-17RC to mediate the IL-17A, IL-17F, or IL-17C ligand response (reviewed by Gaffen 2009). IL-17R binding to ligands and dimerization leads to the recruitment of Act1 through the SEFIR domains, and Act1 is able to bind TRAF3, TRAF6, IKKε, and NEMO resulting in NF-κB activation. Interestingly, Act1 also binds and activates to IRF3, linking IL-17Rs with the antiviral response (Ryzhakov et al. 2012). Interestingly, it has been demonstrated that zebrafish Act1 is able to bind to human IL-17RA and transduce the downstream activation of IRF3 and the antiviral response (Ryzhakov et al. 2012), demonstrating that the pathway is evolutionary conserved. In fact, we already demonstrated the up-regulation or *irf3* in the brain of NNV-infected sea bass (Valero et al. 2015), allowing us to speculate about the link of the Th17 biology with the interferon and antiviral responses. It would be interesting and needed to ascertain the fish IL-17R dimerization affinities and ligand binding, and how they affect to the antiviral response. Our correlation study does not add too many clues on this issue because the correlations are not very high or consistent among the two species or between the receptors and ligands. For example, in sea bass, all the IL-17 receptors are highly correlated suggesting they might be distributed and regulated in a similar way, but not in seabream, which is unlikely. Conversely, low correlations are established between the ligands and receptors making it very difficult to predict the binding specificity.

In conclusion, we have identified for the first time the coding genes for six IL-17 receptors in the teleost fish gilthead seabream and European sea bass revealing the presence and good conservation of the protein domains between fish and human orthologues. IL-17Rs show wide and constitutive transcription in fish tissues, mainly in mucosal tissues such as skin, gills or intestine. In vitro stimulation of HKLs resulted in little regulation, but the transcription induced by T mitogens clearly suggests a T lymphocyte expression. NNV infection in vivo shows that most of the *il17 receptors* in the brain of sea bass, the target tissue, and the susceptible species are up-regulated. Our data suggest that IL-17 receptors might be involved in the antiviral immune response against NNV, probably through T cells, but more efforts are needed to ascertain their role in fish immunity, and in the antiviral response in particular.

### Supplementary Information

Below is the link to the electronic supplementary material.

Supplementary file1 (PDF 164 KB)

Supplementary file2 (DOCX 15 KB)

## Data Availability

Sequences are available in the Ensemb or NCBI databases. Data will be made available upon request.
