# Supplementary material for: Identification and functional characterization of fish IL-17 receptors suggest important roles in the response to nodavirus infection
Source: Mar Life Sci Technol. 2024 Apr 18;6(2):252–65. doi: 10.1007/s42995-024-00225-1 (PMC11136934; doi:10.1007/s42995-024-00225-1)
Supplement: Supplementary file 1 — Supplementary file1 (PDF 164 KB) [file 42995_2024_225_MOESM1_ESM.pdf]

Supplementary Figure S1. Alignment of the putative gilthead seabream and European sea bass IL-17 receptors with their human orthologues. Dashes are introduced to optimize the alignment using the ClustalW software. Residues with >70% similarity are shaded. Percentage of identity (Id) and similarity (Sim) among species is indicated at the end of each sequence.

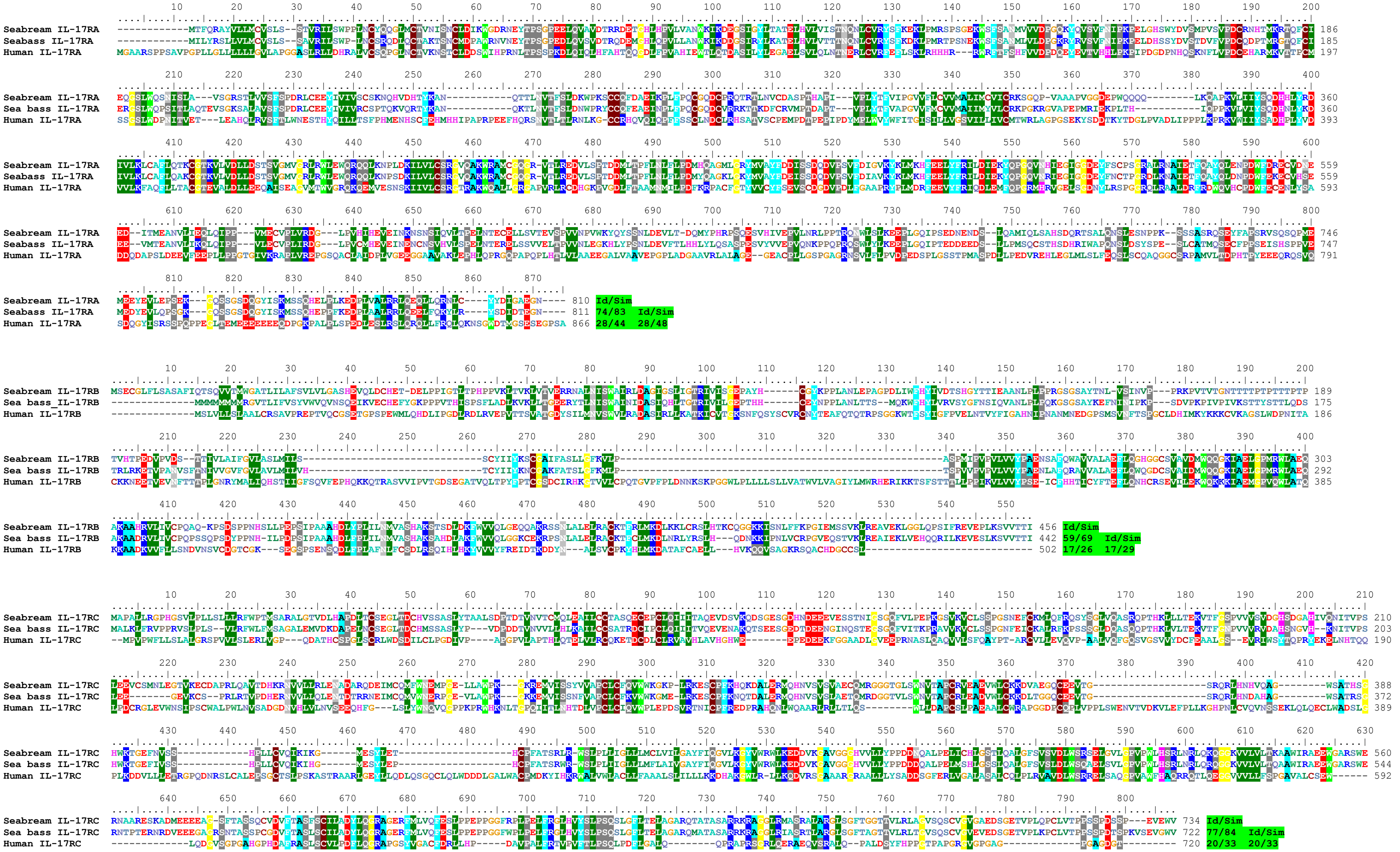

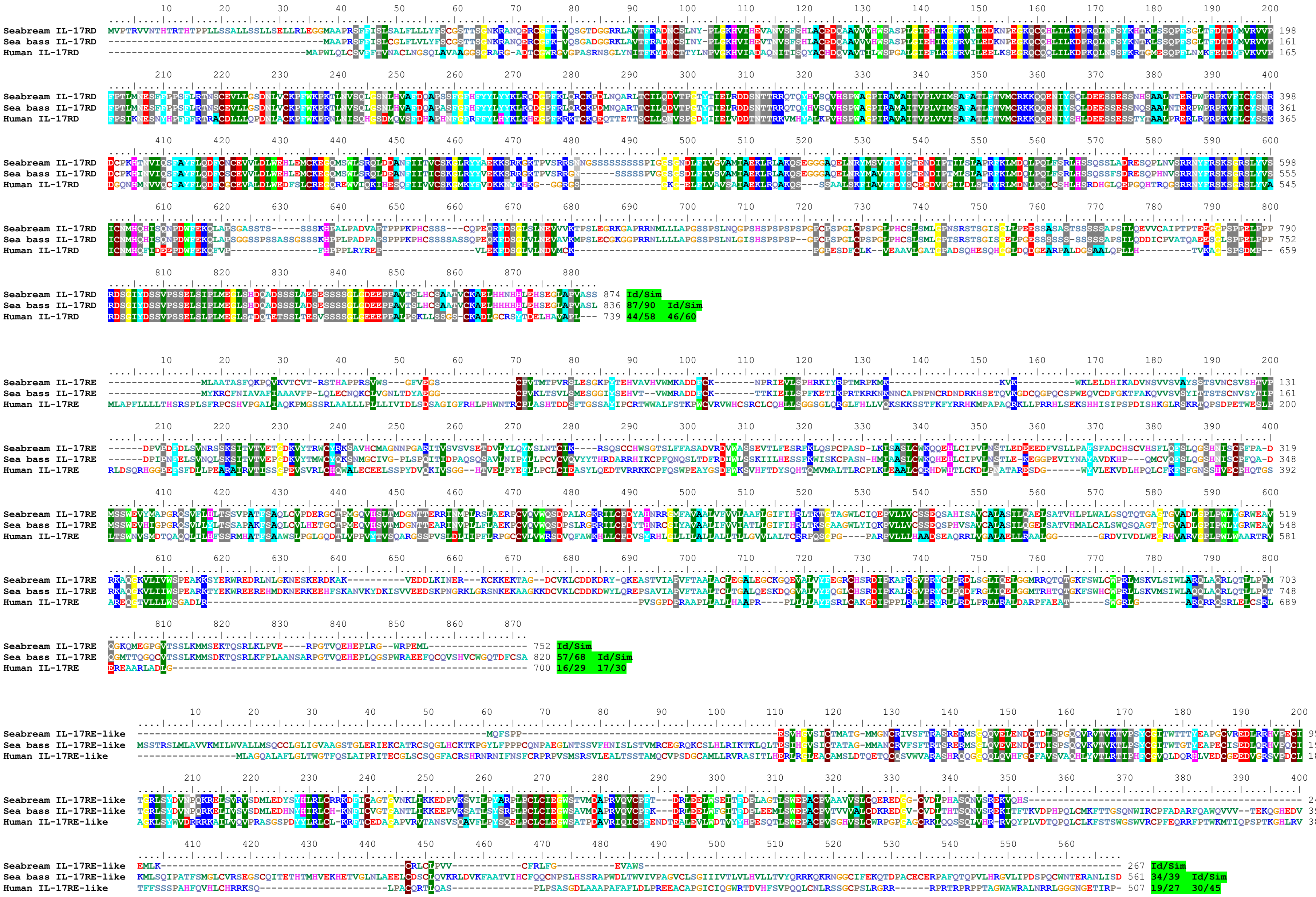
Seabream IL-17RD
Sea bass IL-17RD
Human IL-17RD
874 Id/Sim
836 87/90 Id/Sim
739 44/58 46/60
Seabream IL-17RE
Sea bass IL-17RE
Human IL-17RE
752 Id/Sim
820 57/68 Id/Sim
700 16/29 17/30
Seabream IL-17RE-like
Sea bass IL-17RE-like
Human IL-17RE-like
267 Id/Sim
561 34/39 Id/Sim
507 19/27 30/45
