# Supplementary material for: Identification and functional characterization of fish IL-17 receptors suggest important roles in the response to nodavirus infection
Source: Mar Life Sci Technol. 2024 Apr 18;6(2):252–65. doi: 10.1007/s42995-024-00225-1 (PMC11136934; doi:10.1007/s42995-024-00225-1)
Supplement: Supplementary file 2 — Supplementary file2 (DOCX 15 KB) [file 42995_2024_225_MOESM2_ESM.docx]

**Supplementary Table S**1. Genes and primer sequences used for the real-time PCR.

| **Fish species** | **Gene name** | **Acc. Number** | **Primer sequences (5’→3’)** |
| --- | --- | --- | --- |
| Gilthead seabream | *il17ra* | ENSSAUT00010003704.1 | CAGAGCCCTGAGGAATGCAA  TGGCCTCCATTGTGATGTCC |
|  | *il17rb* | XP_030279211 | AGTGTCAGGGTGGCAAGAAG  AGTCCGCCCAGCTTTTCTAC |
|  | *il17rc* | XM_030421855 | ATCAGCCAGACGGAAAAGGG  GCCAGGCGTAACACAGTAGT |
|  | *il17rd* | XM_030419133 | TCCCAAACACACCAACGTCA  TGCACATCTCCAGGTGTTCC |
|  | *il17re* | OR146493 | TTACAGTCACCGTGTCCAGC  AGTCTTCCTCCTCGTCCTCC |
|  | *il17re-like* | XP_030295872 | TGCTGCCACAGTCAATCACT  CTGGTACAACGACCCAGGTC |
|  | *ef1a* | AF184170 | CTTCAACGCTCAGGTCATCAT  GCACAGCGAAACGACCAAGGGGA |
|  | *rps18* | AM490061 | AGGGTGTTGGCAGACGTTAC  CGCTCAACCTCCTCATCAGT |
| European sea bass | *il17ra* | [ENSDLAT00005079742](http://www.ensembl.org/Dicentrarchus_labrax/Transcript/Sequence_cDNA?db=core;g=ENSDLAG00005035026;r=CAJNNU010000024.1:1671356-1718712;t=ENSDLAT00005079742;tl=A3nplmPcjk4gPGjh-9290461-913905325) | TCCAGTCCTTGAGTGCGTTC  GCTCAGTGTTCAGCTCAGGT |
|  | *il17rb* | [ENSDLAT00005064159](http://www.ensembl.org/Dicentrarchus_labrax/Transcript/Summary?db=core;g=ENSDLAG00005025422;r=CAJNNU010000015.1:20086716-20092775;t=ENSDLAT00005064159;tl=A3nplmPcjk4gPGjh-9290462-913905315) | TGGCTATCGACATGTGGCAG  GCAGACGATGAGCACTCGAT |
|  | *il17rc* | [ENSDLAT00005021273](http://www.ensembl.org/Dicentrarchus_labrax/Transcript/Summary?db=core;g=ENSDLAG00005009295;r=CAJNNU010000015.1:10831976-10841871;t=ENSDLAT00005021273;tl=A3nplmPcjk4gPGjh-9290463-913905311) | CGGCAAATGGCTACAGCATC  GTAGTCCCTGCTGTGAACCC |
|  | *il17rd* | [ENSDLAT00005001775](http://www.ensembl.org/Dicentrarchus_labrax/Transcript/Summary?db=core;g=ENSDLAG00005000676;r=CAJNNU010000011.1:31077422-31105806;t=ENSDLAT00005001775;tl=A3nplmPcjk4gPGjh-9290464-913905337) | CACAGTCCCAGTCCAAGTCC  GACCTTGAAGTGGGTCCCAG |
|  | *il17re* | [ENSDLAT00005021171](http://www.ensembl.org/Dicentrarchus_labrax/Transcript/Summary?db=core;g=ENSDLAG00005009246;r=CAJNNU010000015.1:10823597-10832136;t=ENSDLAT00005021171;tl=A3nplmPcjk4gPGjh-9290465-913905308) | GCTCTGACCTGCCTAACTGG  TCAAGGCCTTTGGGATGTCC |
|  | *il17re-like* | [ENSDLAT00005088326](http://www.ensembl.org/Dicentrarchus_labrax/Transcript/Summary?db=core;g=ENSDLAG00005030239;r=CAJNNU010000024.1:9347889-9365537;t=ENSDLAT00005088326;tl=A3nplmPcjk4gPGjh-9290466-913905305) | TGGATCAGGTGTCCCTTTGC  GGTTGCAGGGATCTGTGACA |
|  | *ef1a* | AJ866727 | CGTTGGCTTCAACATCAAGA  GAAGTTGTCTGCTCCCTTGG |
|  | *rps18* | AY831388 | TTCCTTTGATCGCTCTTAACG  TCTGATAAATGCACGCATCC |
